# How many trials are enough? Familiarization requirements in the Loughborough Soccer Passing Test

**DOI:** 10.1186/s13102-026-01538-7

**Published:** 2026-01-22

**Authors:** Burak Çağlar Yaşlı, Raci Karayiğit, Aysberg Şamil Önlü, Özcan Esen, Gabriel Mareș, Dan Iulian Alexe, Gabriel Stănică Lupu, Cristina Ioana Alexe

**Affiliations:** 1 Department of Coaching Education, Iğdır University, Iğdır, Türkiye; 2 Department of Coaching Education, Ankara University, Ankara, Türkiye; 3 Department of Sport Management, Lokman Hekim University, Ankara, Türkiye; 4 Department of Sport, Exercise and Rehabilitation, Northumbria University, Newcastle-upon-Tyne, NE1 8ST UK; 5 Department of Physical and Occupational Therapy, “Vasile Alecsandri” University of Bacău, Bacău, 600115 Romania; 6 Department of Physical Education and Sports Performance, “Vasile Alecsandri” University of Bacău, Bacău, 600115 Romania; 7 Ankara University Performance Analysis in Sports Application and Research Center (ASPAM), Ankara, Türkiye

**Keywords:** Loughborough soccer passing test, Familirisation, Football, Passing, Skill, Soccer, Female athletes

## Abstract

**Background:**

The Loughborough Soccer Passing Test (LSPT) has proven to be a valid and reliable method for assessing soccer skill performance. However, there are various preparation protocols that exist for the LSPT. The aim of this study is to determine how many trials are required to gather reliable data during LSPT familiarisation.

**Methods:**

81 subelite male youths (12.70 ± 2.67 years; 158.32 ± 17.21 cm; 49.01 ± 14.19 kg) and 17 professional female soccer players (21.47 ± 2.37 years; 164.70 ± 5.00 cm; 57.11 ± 4.27 kg) participated in the study. Following a brief explanation, the participants underwent five repetitions of the LSPT, after which differences between trials were analysed using mixed ANOVA for original time, penalty time, and performance time of LSPT. Adjusted residual values and the Smallest Worthwhile Change (SWC) approach were employed to assess the distributions across repetitions and the practical significance of the observed differences.

**Results:**

Mixed-repeated measures ANOVA showed that Trial 1 significantly underperformed relative to the subsequent four trials across all LSPT values (*p* < .05). There were no statistical differences observed within other trials. Considerable improvement was noted solely between the first trials and subsequent attempts; exceeding the smallest worthwhile change (SWC) thresholds of 1.38 s, 2.29 s, and 3.36 s for original time, penalty time, and total performance time, respectively. Performance time enhanced by 5% between first two trials, however strength of acquisitions remain inconsistent and negligible subsequent trials.

**Conclusion:**

A single trial primarily diminishes the learning effect in LSPT preparation, especially in young sub-elite athletes and professional female athletes. In particular, in situations where samples are large or testing time is limited, implementing a single familiarisation trial seems a practical and efficient option. At the same time, given the higher variability of penalty time, specialists can consider adapting the number of trials depending on the testing objectives. This finding has applicative value for coaches, physical trainers and researchers, as it allows them to reduce the time and resources required for the testing protocol without compromising the validity and reproducibility of the results.

## Background

After Ali Ajmol and colleagues [1] republished measurement properties of the Modified Loughborough Soccer Passing Test (LSPT) in 2007, this paper became a frequently cited reference for the research community (390 citations on Google Scholar and 158 citations on Web of Science as of April 2025). LSPT is shown to be a valid and reliable method of assessing soccer skill performance; numerous studies elucidate the measurement properties and feasibility of LSPT [2–6], but others also examine its use in skill learning [7], practical significance in games [8], its application across various terrains [9], and potential influencing factors [10].

The LSPT procedure involves participants passing the ball 16 times while surrounded by a rectangular bench. Each bench is equipped with a colourful metal strip or a piece of coloured cardboard (0.6 × 0.3 m) that acts as a target area for making effective passes following one of four randomly selected colour sequences. Participants are required to perform the 16 brief passes as quickly and accurately as possible. Time-related metrics are used to calculate LSPT scores, which include original time (the duration taken to complete the 16 passes), penalty time (the amount of time calculated for mistakes, such as incorrect passes, stepping out of the designated area and slow performance), and total time (the sum of original time and penalty time) [1].

Proper administration of any assessment is a crucial part of methodological reliability. To ensure robust and reliable data collection, subjects should become familiar with the procedures [11]. Familiarisation sessions not only ensure that performance changes are not attributable to learning effects [12], but also boost participant interaction and confidence [13], as well as alleviate the effects of hypohydration [14]. While the familiarisation effect has been linked to modified metabolic feedback mechanisms [15], including mental representation of movement [16], synchronisation of motor units [17] and pacing strategy adjustment [18], its effectiveness depends on its structure [19], type of measurement [20, 21], skills complexity [22], and the athlete’s status [23]. Familiarisation is a critical component of field-based performance testing, as early exposures often produce rapid improvements linked to initial motor learning processes [24]. Several sport-specific tests have indicated that even a single practice trial can meaningfully reduce learning effects, while others may require multiple exposures depending on task complexity [19]. Despite its relevance, the LSPT’s familiarisation requirements have not been systematically examined.

In their seminal works, Ali Ajmol et al. [25] emphasised the importance of preparation for LSPT: ‘As with all such tests, participants should be fully familiarised with the protocol before use in an experimental context’ (page 258, right column). They provide participants with five attempts to become accustomed to the protocol prior to the main experiment. From that point on, despite some works continuing in the footsteps of Ali Ajmol and colleagues [8, 26–34], many others prefer different processes during their LSPT preparation [7, 9, 35–38]. The existing evidence demonstrated that practitioners’ preparation methods for LSPT have significantly diverged since its modification [26–50]. While many studies employed two to five trials, others reported as many as ten [4, 6, 38, 45, 46]. Certain studies allocated a single distinct day for familiarisation [31, 32], whereas others prolonged this process over a duration of 4–6 days [7, 29, 43, 44]. Moreover, certain research teams opt to continue until test scores stabilise prior to the main trials [4, 46], whereas others either do not specify or lack a familiarisation process in their methodology [47–50]. It remains unclear what optimal familiarisation should consist of for LSPT. There is currently no published data available for the ideal length of LSPT familiarisation required.

McDermott et al. [39] have reported that familiarity might not be required in all cases, as the initial four LSPT scores for competitive young football players demonstrate consistency across various days. This finding stands in contrast to earlier LSPT research, which highlighted variability in first trials, even with a one-day interval [1]. Furthermore, Lemaol et al. [6] observed that elite players achieve identical results in pre-familiarisation trials 1 and 2, whereas non-elite players do not display the same level of consistency. Despite non-elite players undergoing extensive familiarisation with the protocol, having completed it ten times over a three-week period, there was no statistically significant enhancement in their results. This finding offers a contrasting perspective to earlier recommendations suggesting that four to five attempts are generally adequate for LSPT preparation [1, 4, 25]. Collectively, the current literature presents inconsistent conclusions, and no clear consensus has emerged regarding the optimal familiarisation strategy for the LSPT.

Therefore, the aim of the present study is to determine how many trials are required to gather reliable data during LSPT familiarisation. Addressing this question also offers insight into what the intraindividual variances of the initial LSPT implementation are. It is hypothesised that LSPT performance would increase progressively across the repeated trials, with a consistent rise observed in each of the five repetitions.

## Methods

The study involved 108 soccer players; however, the protocols led to exclusion of ten athletes. The reasons for exclusion included failure to complete the required LSPT trials (*n* = 6), injury (*n* = 2), voluntary withdrawal (*n* = 2), and one case of invalid data. 81 young male footballers (12.70 ± 2.67 years; 158.32 ± 17.21 cm; 49.01 ± 14.19 kg) and 17 professional female soccer players (21.47 ± 2.37 years; 164.70 ± 5.00 cm; 57.11 ± 4.27 kg) were selected from three different football clubs. Female athletes competing in the Turkish Women’s 1st professional football league. Male players were chosen from the U10, U11, U13, U14, U16, and U18 categories from the academies of two distinct local clubs (Table 1). Informed consent was obtained from all players and permission from their parents prior to the commencement of the study for players under 18 years of age. The study was conducted in accordance with the Declaration of Helsinki and its later amendments. The study was approved by the Ethics Committee of Ankara University (22.05.2025 E-79176220-050.04-1828013). Written informed consent to participate was obtained from all participants. For participants under the age of 18 years, written informed consent was additionally obtained from their parent or legal guardian.

**Table 1:** Demographic and training characteristics of participants

Category (*n*)	Positions				Training frequency	Training history (years)
	Goalkeeper	Defender	Midfielder	Forward		
U10 (12)	1	5	4	2	2 day/w	2.1 ± 0.9
U11 (11)	2	4	4	1	3 day/w	2.8 ± 1.0
U13 (14)	-	5	8	1		4.5 ± 1.8
U14 (14)	-	5	7	1		3.0 ± 1.2
U16 (15)	-	4	10	1		4.5 ± 1.6
U18 (15)	2	6	7	-		5.6 ± 2.1
Elite Professional Female (17)	-	5	10	2	5 day/w	8.8 ± 2.8
Total 98	5	34	50	8		4.7 ± 2.8

U = under

**Fig. 1:**
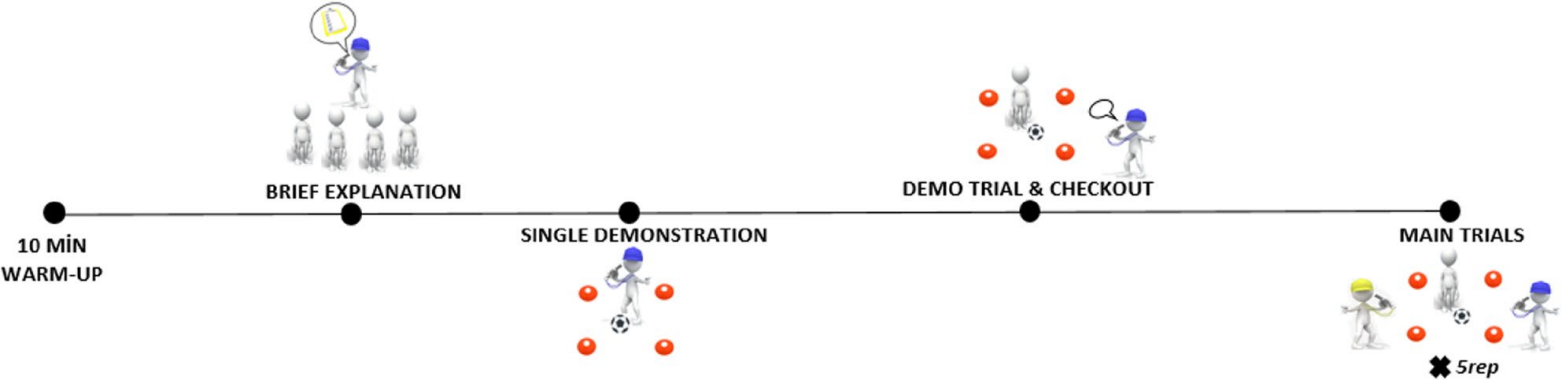
The experimental protocol

On the experimental day, a sequential methodology comprising instruction, demonstration, practice, and feedback (if required) was employed to elucidate the test procedures to participants [24]. Prior to testing, all athletes completed a standardised warm-up lasting approximately 10 min. The protocol began with 5 min of jogging, followed by 3–4 min of dynamic stretching targeting the major lower- and upper-body muscle groups. Dynamic movement drills were then performed, including lateral, diagonal, and backward stepping patterns, along with forward, backward, and medial–lateral leg swings. The warm-up concluded with three short accelerations/sprints. Upon completion of the warm up, participants gathered around the first author in groups of 4 to 5 people, where he provided a preliminary description of the LSPT procedures. During this brief explanation, the primary author outlined the objectives and penalties of the LSPT in a consistent order across all experimental groups, and then he performed the test one time for a single visual demonstration. Following this demonstration, each athlete performed a pre-familiarisation trial consisting of 7–8 passes under testing conditions to rehearse the protocol. If they required any information, they received feedback from testers. Upon concluding the narration process, LSPT was executed in a total of 5 repetitions on artificial turf; these data were documented as research data. The rationale for five repetitions of the selection aligns with the recommended level of familiarity in the original LSPT study [1]. It is also one of the most preferred familiarisation techniques in the literature, along with 2–3 trial procedures; thus, it allows for a comparison of the learning effect between the initial 1–2 repetitions and the 4–5 repetitions. Repetitions were performed in small groups (4–5 athletes), with each group completing one full round before proceeding to the next repetition. During the recovery periods, athletes remained standing or walked slowly within the testing area. No additional stretching or technical drills were permitted. None of the participants had prior experience with LSPT. All tests were administered after 5 p.m., following a cessation of training for at least 24 h. No notable environmental changes occurred across trials. All trials were conducted on the same outdoor artificial turf pitch. Water was provided ad libitum; they were told to drink water if they were thirsty or needed it [1, 51]. All the procedures and tests are implemented with the primary and second authors of this article so as to eliminate inter-experimenter variability. Both authors are former licensed footballers with coaching experience, and they have recently implemented the LSPT more than a hundred times. To evaluate the intra-tester reliability of the first author’s assessments, thirteen athlete tests are recorded with a digital camera, and then the author analyses these recordings twice, separated by two weeks. Although observing the LSPT via video differs from in-field observation, the Intra-Class Correlation Coefficients (ICC 3,1; two-way mixed-effects model, absolute agreement) demonstrated excellent reliability, with values of 0.95 for penalty time and 0.97 for performance time. Figure 1 provides a schematic representation of the experimental procedure.

All statistical analyses were performed using IBM SPSS Statistics *(Version 28*,* IBM Corp.*,* Armonk*,* NY*,* USA).* Mixed-design repeated measures ANOVA was used for data analysis. In the mixed-design repeated-measures ANOVA, Group (7 levels) was included as the between-subject fixed effect, Repetition (5 levels) as the within-subject fixed effect, and the Group × Repetition interaction was also examined. Effect sizes were quantified using partial eta-squared (ηp²). Post hoc pairwise comparisons were conducted using the Bonferroni correction. The normality of the data was assessed using the Shapiro-Wilk test. Normal distribution was observed in the majority of the group by repetition, while only minor deviations were detected in a small number of cases. Parametric tests exhibit robustness to minor deviations from normality when sample sizes are sufficiently large (*n* > 10) due to the Central Limit Theorem [52, 53]. Mauchly’s test of sphericity indicated that the assumption of sphericity was met (*p* > .05), and therefore no correction to the degrees of freedom was applied. Following the mixed ANOVA results, the continuous performance variable was transformed into categorical data based on performance ranking. It provides more information about the dynamics of performance ranking changes over time and how performance levels were distributed across repetitions. Adjusted residual values surpassing the ± 1.96 threshold were regarded as significantly over-represented or under-represented compared to the expected frequencies. These residuals were derived from a chi-square test used to examine differences in the distribution of performance categories across repetitions. Furthermore, to enhance comprehension of variability among repetitions, the Coefficient of Variation (CV%) and Intraclass Correlation Coefficient (ICC) were computed. The CV% was calculated using the standard formula: CV% = (SD / Mean) × 100. The CV values < 5% were considered very low variability, 5–10% low, 10–20% moderate, and > 20% high [54]. The ICC was calculated using a two-way random-effects model with absolute agreement. Both ICC(2,1) and ICC(2,k) were reported because the study aimed to evaluate the reliability of single trials as well as the reliability obtained when multiple repetitions are averaged, offering a more complete picture of LSPT stability during familiarisation. Interpretation followed established benchmarks, whereby ICC values < 0.50 indicate poor reliability, 0.50–0.75 moderate, 0.75–0.90 good, and > 0.90 excellent agreement [55]. To determine whether observed changes between trials were large enough to be practically meaningful, the Smallest Worthwhile Change (SWC) was calculated as 0.2 multiplied by the between-subject standard deviation, in line with previous recommendations [56]. A sensitivity analysis *was performed in G*Power (version 3.1) to evaluate the minimum detectable effect size with the final sample size. With *N* = 98 participants, the design had sufficient power (0.80) to detect medium-sized effects (Cohen’s f = 0.25) at α = 0.05.

## Results

### Original time

**Fig. 2:**
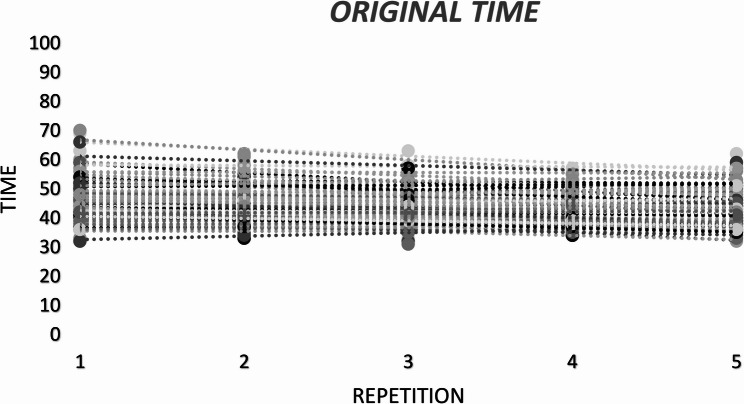
Individual original time scores across five repetitions. Each dot represents a participant's score per repetition

**Fig. 3:**
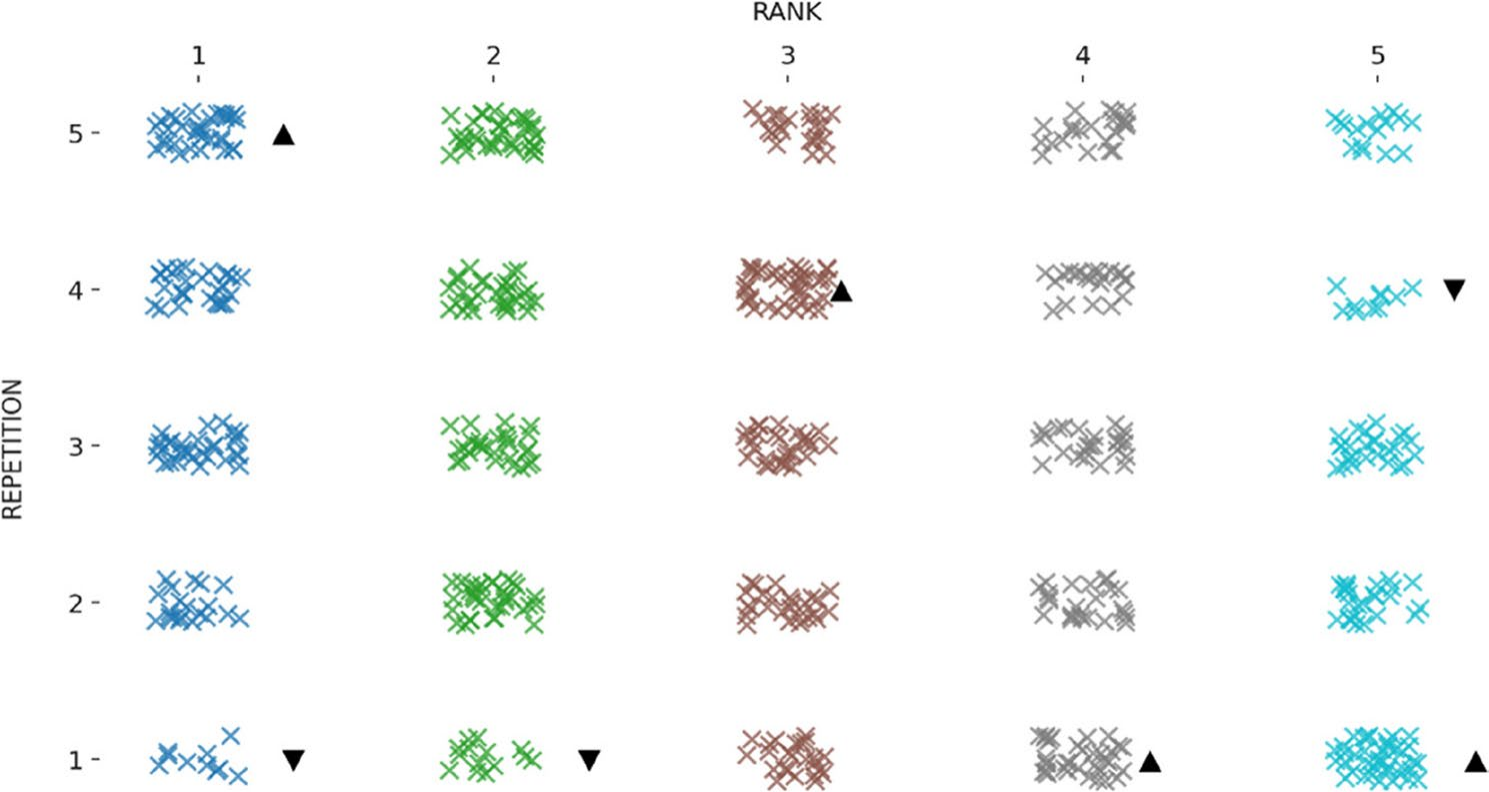
Distribution of original time rankings across repetitions.This dot plot illustrates the distribution of performance rankings (from 1st to 5th) based on original time across five repetitions. Each “×” represents a participant’s ranking in a given repetition. ▲ indicates a repetition where the observed frequency of that rank is higher than expected, while ▼ indicates a repetition where it is lower than expected, based on adjusted residuals (± 1.96)

Table 2; Fig. 2 present the mean original-time scores across the five repetitions. There was a significant main effect of repetition on LSPT original time, *F*(4, 364) = 13.31, *p* < .001, *np*^*2*^ = 0.12, whereas the repetition x group interaction was not significant, F(24, 364 = 1.26, *p* = .184, np^2^ = 0.07. Post-hoc comparisons indicated that the first repetition (M = 47.2 s, SE = 0.57) was significantly slower than the second (M = 45.3 s, SE = 0.46), third (M = 44.5 s, SE = 0.50), fourth (M = 44.1 s, SE = 0.40) and fifth repetitions (M = 44.1 s, SE = 0.43). No other pairwise differences were detected (*p* > .05). The SWC for the original time was 1.38 s. Only the change from Trial 1 to Trial 2 exceeded this threshold; changes between subsequent repetitions were smaller than the SWC (Table 2). Original-time variability across repetitions was low (CV = 7.4%), with moderate reliability for single trials (ICC(2,1) = 0.715) and excellent reliability when averaging multiple repetitions (ICC(2,k) = 0.926). Figure 3 depicts the distribution of performance categories across five repetitions. The distribution differed significantly across repetitions, *X*^*2*^(16) = 62.72, *p < .001.* The best performances were over-represented in the fifth repetition and under-represented in the first repetition, exceeding (± 1.96).

**Table 2:** Descriptive statistics and repeated measures ANOVA results for LSPT variables across five repetitions

LSPT	1.REPS (M ± SD)	2.REPS (M ± SD)	3.REPS (M ± SD)	4.REPS (M ± SD)	5.REPS (M ± SD)	REP		REP * GRP	
						*p*	ηp²	*p*	ηp²
ORIGINAL TIME (s)	46.4 ± 7.9*	44.7 ± 6.5	43.9 ± 7.1	43.6 ± 5.7	43.4 ± 6.5	**0.001**	0.12	0.184	0.07
PENALTY TIME (s)	20.1 ± 12.8*	16.1 ± 11.6	16.2 ± 11.2	14.7 ± 10.2	14.5 ± 10.3	**0.001**	0.08	0.415	0.06
PERFORMANCE TIME (s)	66.6 ± 19.0*	60.9 ± 16.7	60.2 ± 17.0	58.4 ± 14.2	57.9 ± 15.5	**0.001**	0.11	0.456	0.06

^ The descriptive means presented in the table are based on raw data, whereas the values presented in the text are based on Bonferroni comparison

^^ (*) indicate significant differences between repetitions based on Bonferroni-adjusted comparisons (*p* < .05)

.

### Penalty time

**Fig. 4:**
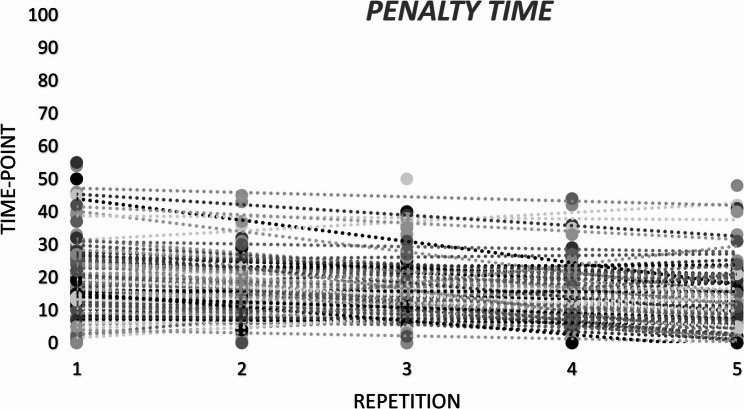
Individual penalty time scores across five repetitions. Each dot represents a participant’s score per repetition. Although penalty time includes subjectively rated error components, the resulting values are expressed in actual time units, allowing for ratio scale treatment

**Fig. 5:**
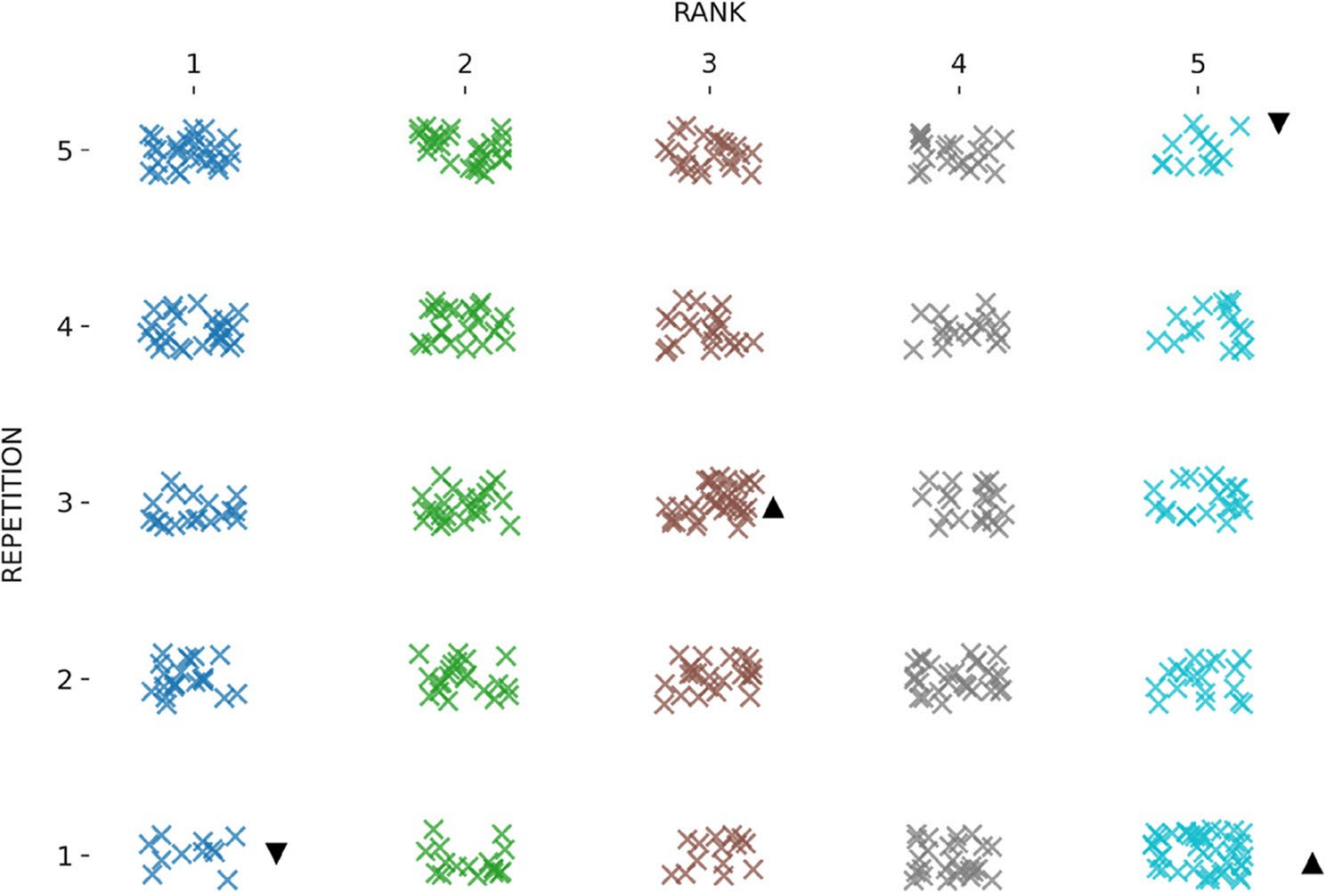
Distribution of penalty time rankings across repetitions. This dot plot illustrates the distribution of performance rankings (from 1st to 5th) based on penalty time across five repetitions. Each “×” represents a participant’s ranking in a given repetition. ▲ indicates a repetition where the observed frequency of that rank is higher than expected, while ▼ indicates a repetition where it is lower than expected, based on adjusted residuals (± 1.96)

Table 2; Fig. 4 present the mean penalty-time scores across the five repetitions. There was a significant main effect of repetition on LSPT penalty time, *F*(4, 364) = 8.39, *p* < .001, *np*^*2*^ = 0.08, whereas the repetition x group interaction was not significant, *F*(24, 364) = 1.03, *p* = .415, *np*^*2*^ = 0.06. Post-hoc comparisons indicated that the first repetition (M = 20.9 s, SE = 1.18) was significantly slower than the second (M = 16.8 s, SE = 1.06), third (M = 16.8 s, SE = 1.06), fourth (M = 15.2 s, SE = 0.96) and fifth repetitions (M = 15.2 s, SE = 0.92). No other pairwise differences were detected (*p* > .05). The SWC for the penalty time was 2.29 s. Only the change from Trial 1 to Trial 2 exceeded this threshold; changes between subsequent repetitions were smaller than the SWC (Table 2). Penalty-time variability across repetitions was high (CV = 56.2%), with poor reliability for single trials (ICC(2,1) = 0.491) and good reliability when averaging multiple repetitions (ICC(2,k) = 0.828). Figure 5 depicts the distribution of performance categories across five repetitions. The distribution differed significantly across repetitions, *X*^*2*^(16) = 44.48, *p* < .001. The best performances were only under-represented in the first repetition, exceeding (± 1.96).

### Performance time

**Fig. 6:**
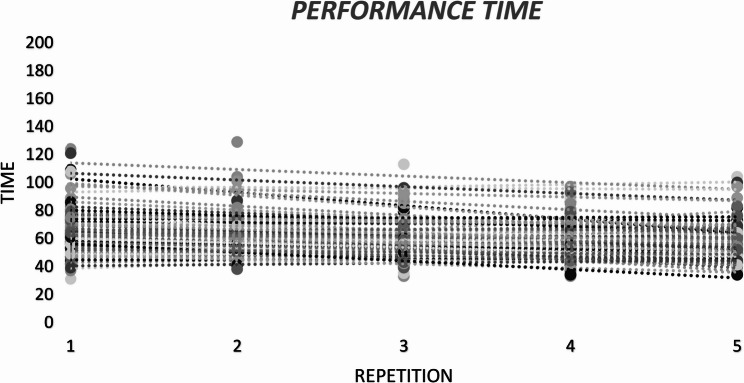
Individual performance time scores across five repetitions. Each dot represents a participant’s score per repetition

**Fig. 7:**
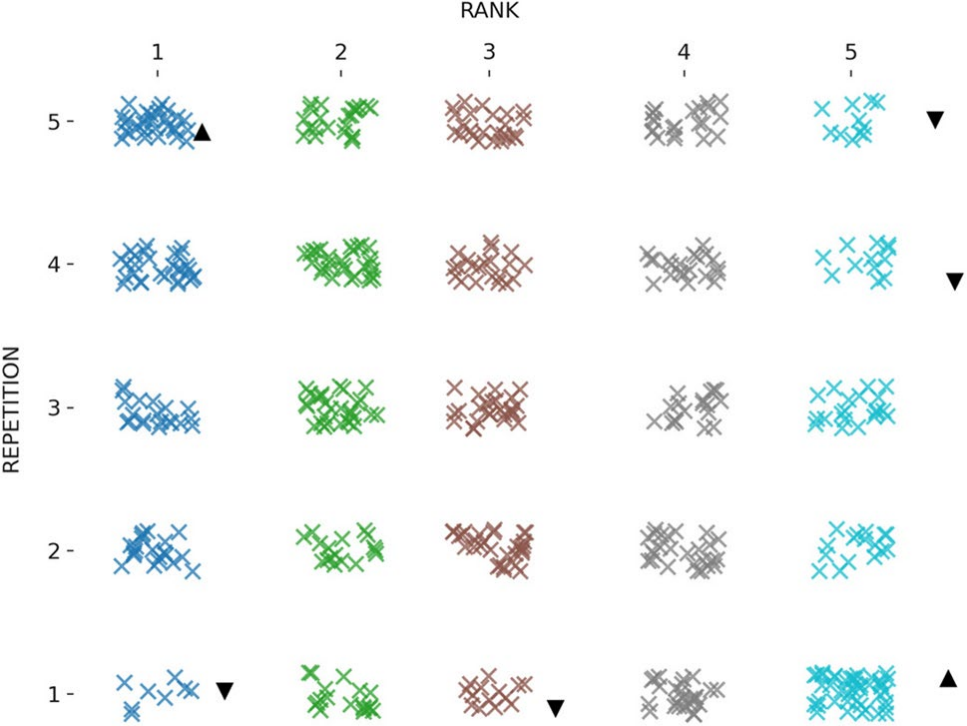
Distribution of performance time rankings across repetitions. This dot plot illustrates the distribution of performance rankings (from 1st to 5th) based on performance time across five repetitions. Each “×” represents a participant’s ranking in a given repetition. ▲ indicates a repetition where the observed frequency of that rank is higher than expected, while ▼ indicates a repetition where it is lower than expected, based on adjusted residuals (± 1.96)

Table 2; Fig. 6 present the mean performance-time scores across the five repetitions. There was a significant main effect of repetition on LSPT performance time, *F*(4, 364) = 12.16, *p* < .001, *np*^*2*^ = 0.11, whereas the repetition x group interaction was not significant, *F*(24, 364) = 1.00, *p* = .456, *np*^*2*^ = 0.06. Post-hoc comparisons indicated that the first repetition (M = 68 s, SE = 1.57) was significantly slower than the second (M = 62.1 s, SE = 1.40), third (M = 61.5 s, SE = 1.43), fourth (M = 59.3 s, SE = 1.22) and fifth repetitions (M = 59.2 s, SE = 1.20). No other pairwise differences were detected (*p* > .05). The SWC for the performance time was 3.36 s. Only the change from Trial 1 to Trial 2 exceeded this threshold; changes between subsequent repetitions were smaller than the SWC (Table 2). Performance-time variability across repetitions was moderate (CV = 15.9%), with moderate reliability for single trials (ICC(2,1) = 0.603) and excellent reliability when averaging multiple repetitions (ICC(2,k) = 0.884). Figure 7 depicts the distribution of performance categories across five repetitions. The distribution differed significantly across repetitions, *X*^*2*^(16) = 62.08, *p <* .001. The best performances were over-represented in the fifth repetition and under-represented in the first repetition, exceeding (± 1.96).

## Discussion

To the best of our knowledge, the current study is the first comprehensive investigation into the LSPT habituation process among young male and professional female football players. Across all LSPT outcomes, Trial 1 consistently demonstrated poorer performance compared to the subsequent repetitions. This suggests that the initial exposure is primarily responsible for the early adjustments in performance, while later trials indicate a more stable pattern of LSPT familiarisation.

These findings may be partly explained by Fitts and Posner’s three-stage learning model [24], although the present study was not designed directly to test motor-learning stages. Athletes might complete the initial cognitive learning stage of the test with a single practice attempt or within a few minutes; this stage is characterised by rapid learning, and improvement may become more stabilised thereafter, consistent with a negatively accelerated learning curve [24]. Similarly, Hopkins et al. [19] reported that performance enhanced by 1.2% between the first two trials but only by 0.2% in subsequent trials, suggesting that at least one practice trial should apply formal physical testing in athletes. Mirkov et al. [57] also reported that a single familiarisation trial was sufficient to produce reliable results in a soccer-specific field test involving throwing, dribbling and kicking skills, and Nuggent et al. [58] found that one familiarisation session is beneficial to minimise the learning effect for knee extension, but not knee flexion during isokinetic testing. Waldron et al. [20] further illustrated a learning effect between tests 1 and 2 in adolescent, but not adult, cyclists. Collectively, previous research reinforces the notion that familiarisation effects in performance tests are typically short-lived [59–61], while their extent and consistency depend on participant characteristics and test spesifics. Further research is warranted to determine whether the pattern observed in this study generalises to different populations or testing environments.

Considering the three LSPT time components (original, penalty, performance), they may to some extent reflect different aspects of skill execution. If these components are viewed along a spectrum of movement precision [24, p.56], original time aligns more closely with gross motor performance, penalty time reflects finer motor control demands, and performance time lies between these two. Accordingly, each metric warrants separate consideration. In the present study, the reproducibility of original time across the first five trials was comparable to values reported in the original LSPT study (CV = 7.4%, ICC = 0.715 & CV = 4.7%, ICC = 0.750), even though those values were obtained after 4–5 familiarisation attempts [1]. This comparison helps contextualise the current findings by showing that original time in our sample reached a similar level of stability with fewer preparatory trials. This may be partly because many athletes are already familiar with the underlying skills, even if they are less accustomed to the specific testing environment. Such characteristics would make this pattern particularly relevant in studies involving large samples or time-limited testing schedules, where a concise familiarisation procedure might offer practical advantage.

The penalty scores exhibit the greatest variability during LSPT familiarisation relative to the original and performance times. This is unsurprising, as tasks requiring finer motor control typically need more exposure for performance to stabilise [22]. Such variability may also reflect the larger number of errors typically observed during the cognitive phase of skill acquisition, particularly when tasks place high precision demands on the performer [24, p.257]. Although penalty-time reproducibility was lower than for the other LSPT components, the average difference between Trial 1 and the subsequent repetitions was still modest (approximately 4–5 s), and the discrepancies among Trials 2–5 were smaller. These patterns indicate that penalty time may require more than a single exposure to stabilise, yet the extent of this requirement remains uncertain. Accordingly, further research is needed to determine the number of repetitions necessary for achieving stable penalty-time performance. Given the higher variability observed, such work may benefit from examining familiarisation protocols extending beyond five repetitions.

The variability of performance time observed in the first five trials of the present study was similar to that reported in elite and non-elite football players [1, 4], despite those values being recorded after 4–5 familiarisation attempts. Only one previous study provides directly comparable data from the familiarisation phase [6], showing that performance-time variability during the first two trials closely resembled our findings but became notably reduced only after an extended period of repeated practice (14 total attempts). The relatively large improvement from Trial 1 to Trial 2, followed by much smaller changes across Trials 2–5, raises uncertainty about whether these modest gains represent meaningful developments or simply normal performance fluctuation. Such variations may also be influenced by individual differences in learning. Given the number of studies examining LSPT performance within the familiarisation phase is limited, further work—particularly with larger samples—would help clarify the stability of performance across repeated attempts and determine whether additional trials beyond the first provide measurable benefit.

## Conclusion

In conclusion, the present findings indicate that the most substantial adjustment in LSPT performance occurs during the first exposure, after which changes become noticeably smaller and more stable. Original and performance times showed relatively consistent patterns across repetitions, whereas penalty time remained highly variable, reflecting the greater complexity of the skills involved. Collectively, these results suggest that one familiarisation trial may be sufficient to minimise the initial learning effect for most athletes—reminding us that additional repetitions do not always translate into meaningful gains. This finding would provide applicative value for coaches, physical trainers and researchers, as it may allow them to reduce the time and resources required for the testing protocol without compromising the validity and reproducibility of the results. The results should nevertheless be interpreted cautiously. The present study examined only five consecutive repetitions and did not assess day-to-day or longer-term variability. Future research should therefore explore whether further stabilisation occurs with a greater number of trials and whether familiarisation requirements differ across age groups, playing levels, or sexes. Given the considerable variability in penalty time, work focusing specifically on this component may help refine familiarisation strategies for the LSPT.

## Data Availability

The original contributions presented in the study are included in the article, further inquiries can be directed to the corresponding author.
